# Multifunctional Roles of Reticular Fibroblastic Cells: More Than Meets the Eye?

**DOI:** 10.1155/2014/402038

**Published:** 2014-04-16

**Authors:** H. G. Alvarenga, L. Marti

**Affiliations:** ^1^Experimental Research, Hospital Israelita Albert Einstein, Avenida Albert Einstein 627, 05152 000 São Paulo, SP, Brazil; ^2^Programa de Alergia e Imunopatologia, Faculdade de Medicina da Universidade de São Paulo (FMUSP), Av. Dr. Arnaldo, 455, 01418 200 São Paulo, SP, Brazil

## Abstract

Fibroblastic reticular cells (FRCs) are stromal cells found in secondary lymphoid organ. Despite its structural function in the lymph nodes being well established, recent studies indicate that the FRCs also play a key role in immunological processes, associated with cell transit, immune response, and cells activation quality, and contribute to peripheral tolerance. To this end, we focus this review on lymph nodes FRC characterization and discuss functional aspects such as production of cytokines and chemokines and their involvement in the immune response, seeking to establish whether certain subsets have a more functional specialization.

## 1. Introduction

Fibroblastic reticular cells (FRCs) are stromal cells of mesodermal origin which are found in secondary lymphoid organs (SLO). The most studied are the FRCs located in the lymph nodes (LN). They comprise distinct subpopulations distributed in the cortical and medullar regions. Some FRCs are found in the T cell zones and were therefore named T cell zone fibroblastic reticular cells (TRCs). The FRCs structural function in the lymph nodes is well established. Yet recent studies indicate that FRCs also participate in the regulation of immune responses and in the maintenance of peripheral tolerance. The cellular traffic in SLO and the activation of specific lymphocyte populations can be influenced by FRCs and consequently also the quality and intensity of the immune responses [1–3]. Nevertheless, this is a relatively new research area and many of the pathways and mechanisms of cellular interactions between FRCs and neighboring cells are not yet known and need further investigation.

The morphological variations among FRCs and the diversity of their functions in the immune response have been described [4–6]. However, so far, a clear match between the described FRC phenotypes and their respective functions has not been established. A further complication to the issue is that mouse FRCs exhibit, in addition to classically known cell surface markers, several other distinct specific markers for FRCs subtypes.

We review herein the studies on phenotypic characterization of FRCs isolated from murine and human lymph nodes and on the selective synthesis of chemokines and cytokines as well as interactions with other cells. Recent advances in understanding the functional diversity of FRC subpopulations are highlighted.

## 2. Fibroblastic Reticular Cells Morphology

**Figure 1:**
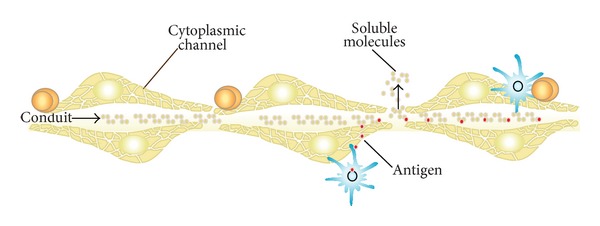
Schematic representation of FRCs. They are spatially arranged so as to delimit a conduit channel that drives soluble molecules. Other structures evidenced are the FRC intracellular cytoplasmic channels, through which antigens are transported from the lymph to the nearby-lying antigen-presenting cells.

Fibroblastic reticular cells (FRCs) are present in the cortical and medullary regions of lymph nodes and can be fusiform, stellate, or highly elongated depending on their localization [7, 8]. Virtually all FRCs contain in their cytoplasm structures consisting of a network of intertwined tubules and cisterns. These complex organelles form labyrinths that open on the cell surface by numerous small orifices, so that the lumen of this tubular system is continuous with the extracellular matrix lattice (Figure 1).

The FRC establishes close contact with each other and also with various other cell types such as lymphocyte, lymphoblast, plasma cell, and interdigitating and follicular dendritic cells (FDC). When FRCs are in contact, their plasma membranes remain separated by a space of about 20 nm, forming an intercellular channel through which driving soluble molecules are transported [8].

More precisely, FRCs organization generates a conduit system between them; these conduits are called reticular fiber network that are responsible for transporting soluble antigens from the afferent lymph to resident dendritic cells in T cell area of the lymph node. This structure produces the infrastructure necessary, at least for the first wave of antigen presentation, which takes place few minutes after soluble antigen injection in a subcutaneous site of an animal model as described by Sixt et al. article [9].

In the lymph nodes cortical areas, FRCs are situated close to the subcapsular sinus, according to their antigen-capturing function. The cortical FRCs are polarized: soluble molecules (antigens, cytokines, neuropeptides, lipids, microbial products) are collected at the FRC cell side facing the subcapsular lumen and released at an area in contact with lymphocytes and follicular dendritic cells (FDC). The intracellular channels of FRCs are the organelles that facilitate the transport of antigen from the afferent lymph to the FDC (Figure 1) [8, 10].

## 3. Phenotypic Diversity

FRC identification is based on a set of markers that are expressed or exclude other cells types. Murine FRCs are basically characterized by the expression of gp38 (a known marker of lymphatic endothelium), absence of CD31 (an endothelial cell marker), as well as the secretion of the chemokines CCL19 and CCL21 and of the cytokine IL-7. However, in the last decade several studies have identified additional markers for these cells present in lymph nodes of different species (Table 1). Although a variety of newer markers may assist the identification of FRCs and their subsets, there is no consensus on the correspondence among the subsets described by several authors.

As an attempt to characterize FRCs in mouse lymph nodes (LN) several fibroblastic cell lines were derived. The cells characterized as FRCs expressed CD44, CD106, and gp38, intracellular ER-TR7, and did not express CD11b/CD18, CD16, CD31, CD32, CD35, LYVE-1, and MHC-II [11].

However it was soon recognized that FRCs are heterogeneous with respect to their morphology and location. Subsets of cells were described in distinct locations of mouse LN, namely, TRC, pericytes and two endothelial subsets BECs (blood endothelial cells) and LECs (lymphatic endothelial cells) that should be taken into consideration, since they need to be excluded when FRCs are the focus of the investigation. Each cell type expresses a distinct combination of the surface markers gp38, CD31, and CD157 to differentiate them [6]. Of note, in this classification, gp38, hitherto considered FRCs hallmark, is not expressed by pericytes and BECs (blood endothelial cells) whereas CD31 is expressed by the endothelial cells (BEC and LEC). The marker CD157 (stromal-like lymphoid marker BP-3) is not expressed by LECs but is present on the other three cell types. However, CD157 was later found to be expressed also by stromal cells in tertiary lymphoid tissues at sites of acute inflammation or tumors [6].

**Figure 2:**
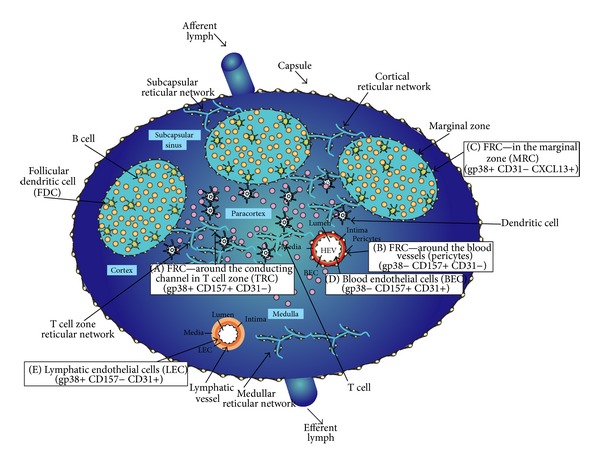
Schematic representation of lymph node and cells classification [4–6]: (A) FRC in the T cell zone (TRC)− gp38+ CD157+ CD31−. (B) FRC around blood vessels (pericytes) gp38− CD157+ CD31−. (C) FRC in the marginal zone (MRC) gp38+ CD31− CXCL13+. (D) Blood vessel endothelial cells (BEC) gp38− CD157+ CD31+. (E) Lymphatic endothelial cells (LEC) gp38+ CD157− CD31+ (E).

Additional studies confirmed that gp38 expression is not unique to mouse TRCs and LECs as it is also expressed by yet another FRC subset found below the LN capsule, known as marginal zone reticular cells (MRCs). Unlike TRCs, the MRCs express CXCL13, MAdCAM-1, and high levels of RANK-L. Detailed studies on the expression of multiple surface markers by the different sets of FRCs were done and the three main FRCs subsets are summarized in Figure 2 [2, 5, 6, 12, 13]. In addition new markers specific for TRCs have been described and compiled [4–6].

Turley et al. and Luther et al. offered yet another classification for mouse FRCs, only the TRCs that differ from the endothelial subsets BECs and LECs [4, 5, 14]. Although the classification was based on gp38 and CD31 expression, other markers such as PDL1 and IFN-inducible iNOS expression were found in TRCs; in addition, the expression of a transcriptional regulator, called deformed epidermal autoregulatory factor 1 (DEAF1), was also present [3, 14, 15].

## 4. FRC in Humans and Primates

Stromal cells from human tonsils with FRC characteristics were also isolated. Tonsils FRCs were compared to bone marrow stromal cells, regarding interactions with B cells. This study showed that both cell types exerted a similar and comparable antiapoptotic effect on B cells. However, after treatment with TNF (tumor necrosis factor)/LT (lymph toxin) either cell type increased the survival of a B cell tumor. While not able to induce purified B cell proliferation, they induced high rates of proliferation in tumor cells [16].

A study on viral pathogenesis in nonhuman primates by Steel et al. identified two other FRC markers besides those described in the mouse by Link et al. [6, 17]. The authors observed that the previously described FRC subsets were positive for a TNF receptor (TNFR) family member known as p75 NGFR and for transglutaminase (TTG) in several species of nonhuman primates. They hypothesized that p75 NGFR expression could be related to a mechanism whereby the nervous system regulates immune responses via FRCs. In addition, they discuss that TTG has previously been shown by Thomazy et al. to play a role in phenotypic regulation of human lymph node FRC [17, 18].

Thomazy et al. compared functional aspects and TTG expression between LN obtained from normal individuals and from lymphoma patients. Basically, when the FRC network is open, migration of cells and molecules around the follicle occurs at high turnover; this condition is accompanied by germinal center expansion and by increased expression of TTG in the FRCs of the subcapsular sinus and cortex. In contrast, when the FRC network is tighter, migration of cells from the subcapsular sinus is reduced and TTG expression is limited to the sinus. In the various lymphomas, high TTG levels were found in the LN stromal cell suggesting that cell migration is altered in these conditions [18].

Not only tumors but also infections with SIV in nonhuman primates or HIV in humans determine the loss of the fibroblast and FRCs network integrity in LN [19].

Fletcher et al. are the sole investigators who made use in humans of murine consensus FRC markers such as gp38, CD31, CD45, CD54, CD106, and PDGFR, to compare the stromal composition between mouse and humans. They reported that the human lymph node stromal cells were marked similar to the murine ones. Using the same murine methods and markers they were able to isolate and cultivate human stromal cell subsets of mesenteric and elsewhere located LN. They found that similar numbers of cells could be isolated from skin-draining and mesenteric LN; however, the stromal composition significantly differed. The FRCs, which grow throughout the T cell zone, were present at greater frequency and were more numerous in skin-draining than mesenteric lymph nodes [20, 21].

Despite the rapid progress in recent years, FRCs characterization is fragmented, and there is still no consensus on the subset markers. In addition, it is not clear whether FRC subpopulations can indeed be classified by the expression of a set of several markers or whether variations in expression are determined by activation status or by their localization in the LN. The definition of FRC phenotypic diversity in murine and human would assess whether these cells have similar morphology combined or not with distinct functions and expression patterns.

## 5. Functional Diversity

Although originally considered as supporting cells in the lymph node, in recent decades it has been suggested that FRC may have additional functions, such as support for lymphocytes migration and survival, activation and control of the immune response as well as a role in peripheral tolerance [21].

Several studies have been conducted in the last decade on the possible functions exerted by FRCs, but a through understanding awaits a clear definition of the existing phenotypic and functional FRC subpopulations. Here, we briefly describe the main functions of the FRCs, focusing on their possible immunomodulatory role on immunity and tolerance.

## 6. FRC Role in Cellular Survival and Migration

Some chemokines can be constantly expressed by FRCs, others maybe expressed at low levels when in idle state and increase the expression in response to stimuli such as TNF-*α*. Others are expressed only after inflammatory stimuli such as TNF-*α*, INF-*γ*, IL-1, or LPS (Table 1).

The reticular network formed by FRCs appears to simultaneously provide mechanical strength to the tissue and space for cell movement, besides acting as a barrier for compartmentalization preventing their disordered interaction or uncontrolled growth [11].

Also, Katakai et al. described a reticular stromal structure in the lymph node cortex, called cortical ridge; the FRCs associated with the cortical ridge may provide a different microenvironment by producing a specialized set of chemokines and adhesion molecules, which would be the attractive destination for immune effector cells in the cortex [22]. Besides, the fibroblastic reticular cell network regulated naive T cell access to the paracortex and also supported and defined the limits of T cell movement within this domain. These data highlight a central role for stromal microanatomy in cell migration within the LN [23].

Another point that supports a role for FRC in cells migration is the interaction between dendritic cells and FRCs. Acton et al. have described that CLEC-2 engagement with gp38 was necessary for DCs to spread and migrate along stromal surfaces and sufficient to induce the membrane protrusions on DCs [24]. Classically the murine FRCs are known to secrete CCL19, CCL21. These molecules have lymphocyte homing properties that can facilitate the cells encounter [4, 5, 10, 19, 25, 26].

The production of these chemokines is important for the T cell movement in the lymphoid organs. The FRCs surrounding the HEV synthesizes CCL21, which interact with intravascular-lying lymphocytes and promote their transmigration through the HEV. Within the LN adhesion molecules present in the stromal cells are thought to facilitate lymphocyte migration along the reticular network [12, 27].

**Figure 3:**
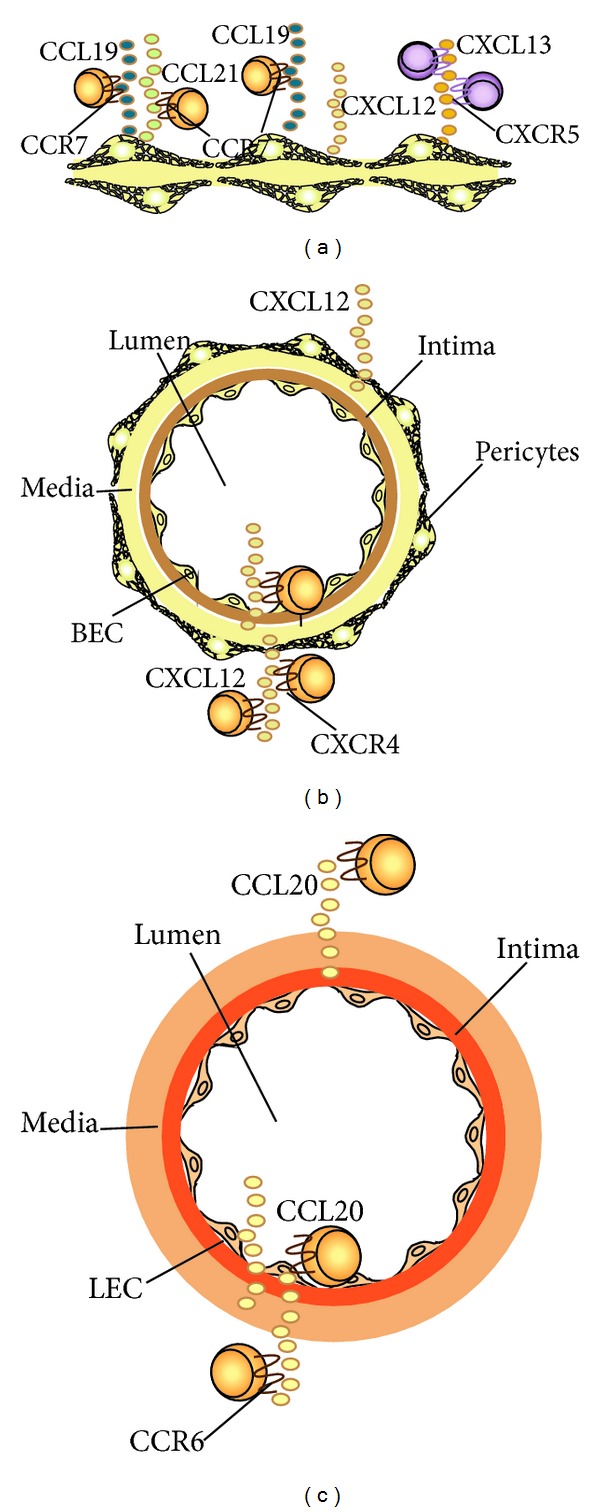
Chemokine production by FRC and endothelial subsets according to Malhotra et al. [28]. (a) FRCs are able to secrete CCL19, CCL21 the ligand of CCR7, CXCL12 ligand of CXCR4, and CXCL13 ligand of CXCR5. (b) BECs express CXCL12 the ligand of CXCR4. (c) LECs express CCL20 the ligand of CCR6.

Malhotra et al. sorted lymph node cells subpopulations and were able to observe that most LECs expressed CCL20, FRCs express large amounts of CCL19 and CCL21, while FRC and BECs expressed CXCL12 only, and FRCs express CXCL13 as shown in Figure 3 [28, 29].

Several groups have reported the cytokine IL-7 as the dominant cytokine synthesized by FRCs. However additional studies have reported production of other cytokines such as IL-6 and IL-15 [4, 5, 11, 17, 27]. Interleukin-7 (IL-7) is a survival factor that acts mainly on naive T cells and also on the development of B cells, DC, and NKT cells [29]. Among its pleiotropic functions IL-7 maintains the T and B cell repertoire and the homeostasis of immune system. TRCs isolated from lymph nodes express ten times more IL-7 than any other lymphoid cell [4].

**Figure 4:**
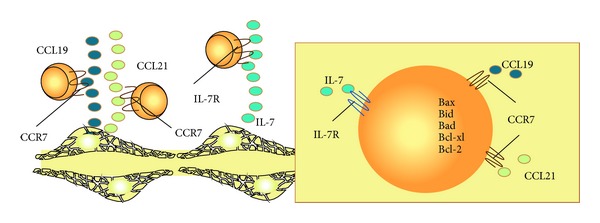
Il-7 in combination with CCL19 and CCL21 leads naive T cell to survival probably by acting in the imbalance of pro- and antiapoptotic proteins.

IL-7 acts in collaboration with chemokines to induce cell survival. Both CCL19 and CCL21 that are also secreted by TRCs are able to induce naive T cells survival (Figure 4). Although presently data suggest that CCL19 and IL-7 are sufficient to maintain T cells survival, it is unclear whether these activities are also shared by other cytokines [4]. Taken together, the results suggest that cell survival maintenance by FRC is not related to a single factor, pathway, or mechanism of action.

During an immune response, within the lymphoid organs, antigen reactive lymphocytes undergo stimulation by the APC, intense proliferation followed by massive (80–90%) cell death as the immune response subsides. The surviving cells differentiate into long-term memory cells that have higher affinity to the antigen. Recent evidence suggests that the lymphoid stromal cell has important roles in both processes, contraction of lymphocyte clones and survival of memory cells. The chemokines CCL19 and CCL21 also promote antigen-induced cell death in activated T cells, suggesting that variations in their relative concentration in the microenvironment may favor either cell death or cell survival. It is also of note that constant stimulation or high doses of CCL19 and CCL21 inhibit the activation, proliferation, and function of T cells [27].

It is important to note that the expression profile of cytokines and chemokines is different between the mesenteric and skin-draining LN-derived FRC. Mesenteric LN-derived FRCs contain reduced expression of several important cytokines and chemokines such as IL-6, IL-7, BAFF, CXCL9, CXCL10, IL-1 receptor accessory protein (IL1RAP), activin receptor IIA, VEGF-A, LIF, and cKIT-ligand [20, 21].

## 7. Immune Cell Recruitment and Activation

The support network by stromal cells existing in lymphoid organs simultaneously provides mechanical resistance and spaces for immune cells active migration [25]. Conversely the reticular network requires a close and continuous contact with immune system cells [11].

The reticular network and the FRC participate in the selection of antigens that enter the subcapsular sinus. High molecular weight molecules are unable to enter the FRCS conduits lumens and are trapped in the subcapsular sinus macrophages. Small molecular weight antigens are carried via the conduits to the other pole of the cell to be released and captured by APCs and activate the Ag-reactive T cells. Roozendaal et al. have clarified that the small molecular weight antigens drain passively into the B cell zone through a follicular conduit system that connects the subcapsular sinus with follicular dendritic cells area. Moreover, the follicular conduit network contains CXCL13. Thus, the conduit system provides a source of antigen as well as a possible pathway or network for guiding B cells to the antigen inside the follicles in a similar manner as identified in the paracortex for T cells [30].

**Figure 5:**
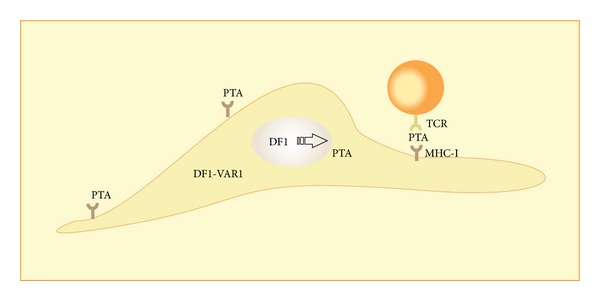
While the expression of AIRE by stromal cells is still a controversy, Turley et al. [14] suggest a role for DF1 and DF1-VAR1 isoforms of DEAF1, on PTAs expression by FRC and the peripheral homeostasis.

IL-7 secreted by FRC promotes APC function and the molecules CCL19 and CCL20 enhance the interaction between dendritic cell (DC) and T cells and stimulate endocytosis and DC antigen presentation (Figure 5). Activated DCs in turn produce CCL3 and CCL4, recruit rare T cell through the FRC driven traffic, and help the immune response priming. All these activities favor the recognition and activation phases of the immune response [5, 12, 27].

Certain molecules, like retinoic acid and vitamin A, are synthesized only by stromal cells from mesenteric LN. The microenvironment appears to influence certain T cell phenotypes. In fact, activated T cells at this location present a gut-homing phenotype since they express *α*4*β*7 and CCR9. Mesenteric LN stromal cells are also better at inducing IgA responses. These data suggest that stromal cells can drive the type and quality of immune response in SLO [12].

In addition, Chai et al. have demonstrated that FRC-deficient animals exhibited an impaired resistance to viral infection, demonstrating that LT*β*R-mediated FRC maturation is critical for the maintenance of the immunocompetence [31].

Another study provides a complete analysis of FRCs number, phenotype, and function associating them with antigen-specific T cell response over time. This study also demonstrates that FRCs are activated in a process dependent on naive lymphocyte cell number trapping induced by dendritic cells [32].

It is noteworthy that FRC can upregulate not only the migration but also T cells homeostasis, and there are signs that they also influenced T cell differentiation in effector and memory cells. However new investigations are required to establish most of these findings [33].

Together, these data suggest that FRC can contribute to the immune response through the cell traffic regulation in lymph nodes, lymphocyte survival, increasing the antigen presentation quality and possibly driving the type of immune response.

## 8. Peripheral Tolerance

FRC has been implicated in peripheral tolerance. Some stromal cells express antigens from several peripheral tissues (PTA) and are involved in maintaining peripheral tolerance, especially by deletion of self-reactive T cells [33–37].

The FRCs expressing epidermal deformed autoregulatory factor 1 (DEAF1), transcription factor encoded by DF1 gene, is responsible for the PTA expression. The DEAF1 controls expression of approximately 300 genes in pancreatic lymph nodes, of which 75% are classified as PTA. Although autoimmune regulator (AIRE) and DEAF1 are similar, they seem to have different manners of action. DEAF1 may have opposing effects on gene transcription in distinct cells [14].

In NOD mice there are at least two DF1 isoforms, canonical DF1 and a DF1 splicing variant (DF1-VAR1). In contrast to DF1, the DF1-VAR1 is unable of entering into the nucleus and regulates PTA transcription (Figure 5). However, it can bind to the DF-1, sequestering it in the cytoplasm and restricting PTA expression. The functional implication of this phenomenon is not yet clear and recent studies suggest that AIRE and DF1 might be inversely expressed in the different stromal populations [20, 21].

The AIRE expression in FRCs is still controversial: while several studies reported AIRE expression in stromal cells, but not specifically in FRC, Siergert et al. reported AIRE expression specifically in FRCs at low levels [20, 25, 34].

The FRC can not only induce tolerance in T cells by the expression of self-antigens but can also limit the T cell response to foreign antigens by expression of suppressive factors leading to direct inhibition of T cells or indirect inhibition by reducing the dendritic cells immunogenicity [33].

In addition to FRC involvement in peripheral tolerance through the self-antigens expression, FRCs are highly responsive to IFN-*γ*. In response to IFN-*γ*, FRC increases NOS2 gene transcription encoding the inducible enzyme nitric oxide synthase (iNOS), resulting in nitrite production, which can block T cell cycle progression. These observations place the FRCs as regulators of T cell activation through direct contact with IFN-*γ* producing T cells [33].

Th2 cells are insensitive to the FRC antiproliferative effect due to a lack in IFN-*γ*. This is a possible mechanism of maintaining the integrity of lymphoid tissue, given the ability of Th1 to cause more tissue damage when compared to Th2 [15].

IFN-*γ* is an important functional regulator of FRC, mainly by the ability of inducing iNOS, IDO-1, and PD-L1 and several molecules involved in antigen presentation. IFN-*γ* is usually considered the main proinflammatory cytokine, acting in various aspects of the immune response such as depletion of intracellular pathogens by macrophages activation, and increased expression of MHC molecules. However, IFN-*γ* may also act in immune-regulatory functions, modulating negatively the expression of proteolytic enzymes, reducing recruitment of inflammatory cells, inhibiting Th17 differentiation, and positively regulating the differentiation of regulatory T cells in certain circumstances. Other proinflammatory cytokines such as TNF-*α* and IL-1*β* produced by activated DC also lead to the induction of iNOS in FRC [33, 34].

Siegert and Luther suggest that the inhibitory role of FRCs engages at least two soluble factors, iNOS and cyclooxygenase (COX-1 and COX-2). COX-2 is the rate-limiting enzyme in the generation of prostanoids including prostaglandin E2 (PGE2), which can either increase or suppress T cell immunity [33].

The effect of FRC in T cell immunity has also been related with PD-L1 expression, which leads to CD8+ T cells modulation. PD-L1 or IDO expression can be induced by IFN-*γ*. In addition, PD-L1 and IDO play important roles in the development of regulatory T cells and myeloid suppressor cells, which can connect FRC with production, maintenance, or activation of regulatory cells [33].

Many cell types are subtypes of the immune system that suppress or regulate adaptive immunity, such as regulatory T and B cells, dendritic cells, and regulatory M2 activated macrophages [33]. So, it is not surprising that FRCs are positive and negative regulators of adaptive immunity. Similar to other cell types, it remains to be clarified whether there is a subtype of FRC dedicated to this function, or if this feature is dependent on their location, activation state, or phase of the immune response.

In this context, structural cells may act as mechanical and chemical sensors of T cells response in inflamed organs and regulate the populations' dynamics to ensure the maintenance of functional structures.

## 9. Conclusion

It is clear that the role of FRC is not restricted to structural support, but that they deeply contribute as immune response regulators. However, new studies that investigate their subpopulations and functions related are necessary, as well as differences between different lymph node localization. Most studies dedicated to this subject were developed in murine, and studies in humans that corroborate these findings are required. Another shortcoming of this knowledge is how and much the FRCs may contribute to the development and/or maintenance of inflammatory diseases, autoimmune diseases, or cancer.

## Figures and Tables

**Table 1:** FRC characterization markers. FRC was classified by species, surface or intracellular makers, inducible markers and ones that exclude other cells presence, and chemokines and cytokines secretion, listed in time order of publishing.

Publication year—author (species)	Markers				Chemokines		Cytokines
	Surface	Intracellular	Inducible	Excluding	Regular	Inducible	
2004—Katakai et al. (a) [11] (MLN)	CD44, gp38, CD106	ER-TR7	CD54	CD11b/CD18, CD16,CD31, CD32, CD35, MHCII, LYVE1	CCL2, CXCL12, CX3CL1	CCL4, CCL5, CCL20, CXCL10	IL6, IL7, IL15
2004—Katakai et al. (b) [22] (MLN)	—	ER-TR7	—	—	—	—	—
2005—Sixt et al. [9] (MLN)	gp38, *α*SMA, desmin	ER-TR7	—	—	—	—	—
2006—Bajénoff et al. [23] (MLN)	Desmin, CD106, CD54	ER-TR7	—	—	CCL19, CCL21, CXCL12 (SDF-1)	—	—
2006—Hara et al. [25] (MLN)	gp38, CD106	ER-TR7	—	—	—	CXCL16	IL7, IL15
2007—Amé-Thomas et al. [16] (HT)	CD73, CD90, CD105	—	CD54 CD106	CD21, CD23, CD35, CD45	CCL5, CXCL9, CXCL10, CXCL12	CCL19	—
2007—Link et al. [4] (MLN)	gp38, *α*SMA, desmin, CD157, PDGFR*α*, PDGFR*β*, CD54, CD106, LT*β*R, TNF-R	ER-TR7	—	CD21, CD31, CD35 CD45, LYVE-1	CCL19, CCL21	—	IL7
2009—Roozendaal et al. [30] (MLN)	gp38	ER-TR7	—	—	—	—	—
2009—Mueller and Germain [12] (review)	gp38, PDL1	VEGF, ER-TR7	—	CD45, CD31, CD21, CD35, C4, CD16, CD23, CD32, CD157, Mfge8	CCL19, CCL21	—	IL7
2009—Steel et al. [17] (NHPLN)	gp38, p75NGFR, TTG, CD54, CD106, CD157, LT*β*R, PDGFR*α*, PDGFR*β*, *α*SMA, TNFR1, Meca79, desmin	ER-TR7	—	—	CCL19, CCL21, CXCL16, CCL2/MCP1	—	IL7, IL6
2010—Turley et al. [14] (review)	gp38, PDL1	DEAF1		CD31	CCL19, CCL21	—	IL7
2011—Khan et al. [15] (MLN)	gp38, PDL1	—	iNOS	CD45, CD31	CCL19, CCL21	—	IL7
2011—Luther et al. [5] (review)	gp38, CD54, CD106, CD157, PDGFR*αβ*, LT*β*R, TNFR1, desmin, *α*SMA	VEGF	—	CD21, CD31, CD35, CD45, C4, Mfge8	CCL19, CCL21	CCL2, CCL4, CCL5, CXCL12, CXCL16, CX3CL1	IL7, IL6
2011—Link et al. [6] (MHLN)	gp38, CD54, CD106, CD157, PDGFR*αβ*, LT*β*R, desmin, *α*SMA	ERTR7	—	CD31, CD35, CD45	CCL19, CCL21	—	IL7
2011—Onder et al. [3] (MLN)	gp38	—	—	CD31, CD45	CCL19, CCL21	—	IL7
2011—Fletcher et al. (a) [20] (MHLN)	gp38, PDGF*α*, CD54, CD106	VEGF	—	CD45, CD31	CCL19, CCL21 CXCL9, CXCL10	—	IL7
2011—Fletcher et al. (b) [21] (review)	gp38, PDGF*α*, CD54, CD106	VEGF	—	CD45, CD31	CCL19, CCL21, CXCL12 (SDF1)	—	IL7
2011—Frontera et al. [37] (MLN)	CD54, CD106, PDGF*α*, CD141	JAMC	—	CD45, CD31, LYVE1	CCL21	—	—
2011—Siegert et al. [2] (MLN)	gp38	iNOS	—	CD45, CD35, CD31, EpCAM	CCL19, CCL21	—	IL7
2011—Lukacs-Kornek [34] (MLN)	gp38, PDL1, INFGR1, TNFR1, TNFR2	NOS2, IDO	—	CD45, CD31	CCL19, CCL21	—	—
2012—Zeng et al. [19] (NHPLN)	Desmin	—	—	CD35, CD21	—	—	—
2012—Siegert and Luther [33] (review)	gp38, PDL1	COX2, Aire, DEAF1, NO	iNOS, MHCII, IDO, CD80	CD21, CD35, CD31	CCL19, CCL21	—	IL7
2012—Graw and Regoes [26] (MLN)	—	—	—	—	CCL19, CCL21	—	—
2012—Onder et al. [29] (MLN)	gp38	—	—	CD31, CD45	CCL19, CCL21	—	IL7
2012—Hess et al. [13] (MLN)	gp38, CD106, MadCAM1	RankL	—	CD31, CD45	—	—	IL7
2012—Malhotra et al. [28] (MLN)	gp38, CD140a	VEGFA and C, ANGPTL2 and 4, HGF, GREM1, SERPINF1 cadherin-11, IFITM-1, Flt3L	—	CD31, CD45	CXCL14, CCL19, CCL21, CXCL13 CXCL12, CCL2, CCL7		IL34
2012—Acton et al. [24] (MLN)	gp38	—	—	CD31	CCL19, CCL21	—	—
2013—Chai et al. [31] (MLN)	gp38, *α*SMA,	ER-TR7, NO	—	CD31, CD45	CCL19, CCL21	—	IL-7
2014—Yang et al. [32] (MLN)	gp38, PDGFR*αβ*, LT*β*R, *α*SMA	VEGF, iNOS, VEGF, MyD88	—	CD31, CD45, LYVE-1	CCL19, CCL21	—	IL-7

Murine lymph node (MLN); human tonsils (HT); nonhuman primate lymph node (NHPLN); murine and human lymph node (MHLN).
